# ﻿Scuttle flies (Diptera, Phoridae) collected by mosquito trap from Košice Zoo, Central Europe

**DOI:** 10.3897/zookeys.1244.146861

**Published:** 2025-07-04

**Authors:** Bernd Grundmann, Katarína Loziaková Peňazziová, Tomáš Csank, Laura Mlynárová, Patrik Pastorek, Jozef Oboňa

**Affiliations:** 1 Diekstraße 6, D – 33824 Werther (Westf.), Germany Unaffiliated Werther Germany; 2 Department of Microbiology and Immunology, University of Veterinary Medicine and Pharmacy, Komenského 73, SK – 04181 Košice, Slovakia University of Veterinary Medicine and Pharmacy Košice Slovakia; 3 Department of Ecology, Faculty of Humanities and Natural Sciences, University of Prešov, 17. novembra 1, SK – 08116 Prešov, Slovakia University of Prešov Prešov Slovakia; 4 ZOO Košice, Ulica k Zoologickej Záhrade 1, SK – 04001 Košice, Slovakia ZOO Košice Košice Slovakia

**Keywords:** Faunistic, mosquito BG-Sentinel 2 trap, new records, Phoridae, Slovakia

## Abstract

In this paper the results of a Phoridae collection from Košice Zoo in eastern Slovakia are reported. An unusual trap method was employed – BG-Sentinel 2 mosquito traps combined with CO_2_ as an attractant. Between July and October 2023, 73 species of the Phoridae family were recorded, 24 of which are new to the Slovak fauna (1 species from the genus *Woodiphora* and 23 species from the genus *Megaselia*). As a result, the total number of Phoridae species documented in Slovakia has increased to 254.

## ﻿Introduction

Phorid flies (Diptera: Phoridae) belong to the suborder Cyclorrhapha, a superfamily within Platypezoidea, comprising approximately 35 genera and more than 700 species in Europe (Oosterbroek, 2006). To date, 230 species have been recorded from Slovakia (Mocek 1997, 2009; Jančík and Disney 2020; Grundmann et al. 2023, 2024). These flies, commonly known as scuttle flies, are characterised by their rapid, somewhat abrupt movements. Adult flies are typically small to medium-sized (0.5–6 mm) and often exhibit a somewhat curved, robust body form (Disney 1983). Most adults feed on nectar, honeydew, sap from fresh carrion, and faeces; some also feed on the body fluids of live beetle larvae and pupae.

According to Disney (1994), Phoridae can be collected by using a wide variety of insect trapping techniques. A commonly employed method is the use of Malaise traps (e.g. Grundmann and Kappert 2023; Caruso et al. 2024). Other methods, such as emergence traps (Szadziewska 1977; Büchs 1988), white and yellow water traps, pitfall traps (Disney et al. 1981), and light traps (Brown and Marshall 1984) are also frequently used. Breeding scuttle flies from natural substrates such as humus, compost, mushrooms, and carrion is another effective method (Disney and Evans 1982; Disney 1994). However, to the authors’ knowledge, mosquito traps have not yet been used to capture scuttle flies.

## ﻿Materials and methods

### ﻿Sampling

**Figure 1.**
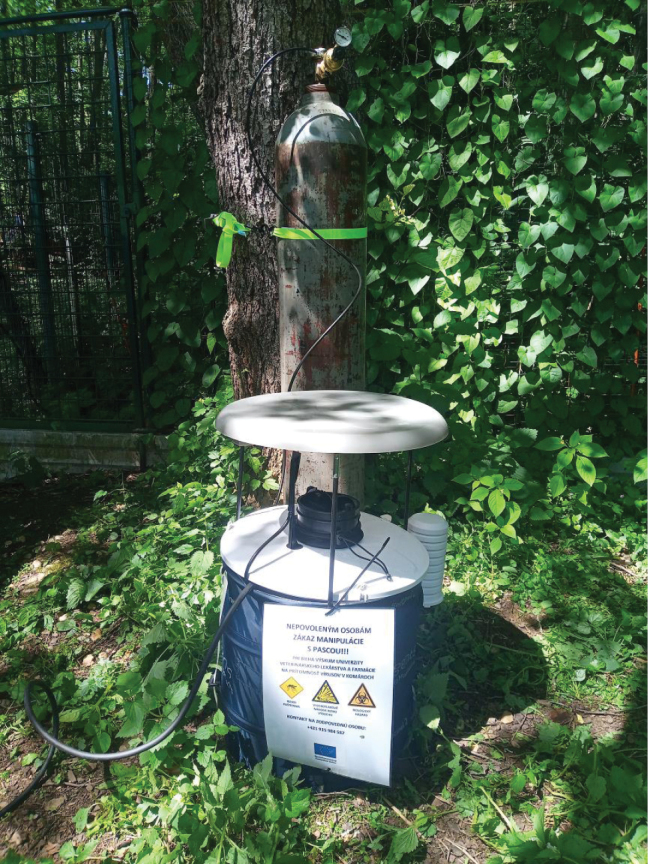
Photograph of BG-Sentinel 2 trap (Biogents, Germany) from Košice Zoo.

A fatal case of West Nile virus infection in a Great Grey Owl (*Strixnebulosi* Forster, 1772) kept at Košice Zoo was diagnosed (Peňazziová et al. 2021). Based on this, mosquito capture was conducted using BG-Sentinel 2 traps (Biogents, Germany) with CO_2_ cylinders as an attractant (Fig. 1). The traps were placed near small lakes in the locality (see locality data) and operated continuously from July 2023 (with the first collection on 17 July) until the end of October 2023 (with last collection on 28 October). The capture nets were replaced twice a week and stored at -20 °C until transport to the laboratory, where they were stored to -80 °C. After sorting the mosquitoes, the remaining material was preserved in 75% alcohol. The collected flies were sorted to family level and identified to species level by BG using identification keys (Schmitz, 1941, 1943, 1951, 1956; Disney, 1983, 1989, 1994, 1999), who also curated the collection.

Locality data:

Slovakia, Košice district, Košice Zoo,

Horný rybník – Upper pond (HR): 48°47'11.4"N 21°12'11.9"E, 412 m a.s.l.

Dolný rybník – Lower pond (DR): 48°47'20.6"N 21°12'22.0"E, 411 m a.s.l.

An asterisk (*) before the species name indicates a new geographical record for Slovakia. References to the online GBIF page resources (with a brief comment on their European distribution) are added for each species. For species new to the Slovak fauna, additional information on their known distribution and the feeding ecology of their larvae (if available) is also included.

## ﻿Results

A total of 487 scuttle flies were collected in the BG-Sentinel 2 traps (July – 100 individuals, August – 96 inds, September – 180 inds, October – 111 inds) in 2023, representing 73 species (see Annotated list of recorded species). Thirty individuals were indeterminate females of the genus *Megaselia* and six individuals belonged to three new species . Twenty-four species were recorded for the first time in Slovakia, and six species represent the second record for Slovakian fauna. The most abundant species were *Megaseliaangusta* (Wood, 1909) with 69 individuals, *Megaseliaframeata* Schmitz, 1927 with 65 inds, and *Megaseliaaculeata* (Schmitz, 1919) with 41 inds. Twenty-seven species were recorded based on only one individual.

### ﻿Annotated list of recorded species

#### ﻿Diptera

**Family: Phoridae**

***Anevrinathoracica* (Meigen, 1804)**

**Material examined.** DR 09.10. ♂.

**GBIF records.** In Europe this species is mainly recorded in the northern regions and only rarely in Central Europe. https://www.gbif.org/species/1545916.

***Coniceradauci* (Meigen, 1830)**

**Material examined.** DR 23.10. ♂; HR 22.08. ♂, 28.08. ♂.

**GBIF records.** In Europe this species is primarily recorded in the northern regions. https://www.gbif.org/species/1546041.

***Conicerafloricola* Schmitz, 1938**

**Material examined.** DR 31.07. ♂, 28.08. ♂, 11.09. ♂, 26.09. ♂; HR 04.09. ♂, 07.09. ♂, 22.09. ♂, 03.10. ♂.

**GBIF records.** In Europe this species is mainly recorded in the northern regions and only sporadically in Central Europe. https://www.gbif.org/species/1546039.

***Coniceratibialis* Schmitz, 1925**

**Material examined.** DR 04.09. 2 ♂♂, 07.09. 2 ♂♂, ♀, 11.09. ♂; HR 31.07. ♂, 11.09. ♂.

**GBIF records.** In Europe this species is mainly recorded in the northern regions. https://www.gbif.org/species/1546012.

***Diplonevranitidula* (Meigen, 1830)**

**Material examined.** DR 03.08. ♀, 09.08. ♂, 11.08. ♂, 04.09. 2 ♂♂, ♀, 13.10. ♂, ♀.

**GBIF records.** In Europe this species is mainly recorded in the northern regions and only sporadically in Central Europe. https://www.gbif.org/species/1548598.

***Gymnophoraarcuata* (Meigen, 1830)**

**Material examined.** DR 31.07. ♂, 09.10. ♂; HR 22.08. ♀.

**GBIF records.** In Europe this species is mainly recorded in the northern regions and only sporadically in Central Europe. https://www.gbif.org/species/1549517.

***Pseudacteonformicarum* (Verrall, 1877)**

**Material examined.** DR 17.07. ♂, 26.07. ♂.

**GBIF records.** In Europe this species is rarely recorded only the northern regions. https://www.gbif.org/species/1549197.

***Triphlebaantricola* (Schmitz, 1918)**

**Material examined.** HR 18.09. ♀.

**GBIF records.** In Europe this species is sporadically recorded in Central Europa and in the northwest of the Mediterranean region. https://www.gbif.org/species/1549712.

***Triphlebadistinguenda* (Strobl, 1892)**

**Material examined.** HR 22.08. ♂.

**GBIF records.** In Europe this species is mainly recorded in the northern regions and only sporadically in Central Europe. https://www.gbif.org/species/1549634.

***Triphlebadudai* (Schmitz, 1918)**

**Material examined.** HR 28.08. ♂, 03.10. ♂.

**GBIF records.** There are only a few records from Sweden. https://www.gbif.org/species/1549630.

***Triphlebalugubris* (Meigen, 1830)**

**Material examined.** DR 09.10. 3 ♂♂.

**GBIF records.** In Europe this species is mainly recorded in the northern regions. https://www.gbif.org/species/1549637.

***Triphlebanudipalpis* (Becker, 1901)**

**Material examined.** HR 21.07. ♂.

**GBIF records.** In Europe this species is mainly recorded in the northern regions and only sporadically in Central Europe. https://www.gbif.org/species/9748380.

*Woodiphoraretroversa (Wood, 1908)

**Material examined.** HR 26.07. ♂.

**Published records.** Distribution in Britain, Denmark (mainland), Hungary, and the Netherlands, with records from Poland (Durska 2013).

**GBIF records.** Additional distribution in Norway. https://www.gbif.org/species/1546062.

**Comments.** This is a summer species, most abundant in July. Its development occurs in carrion (Schmitz 1956).

***Megaseliaaculeata* (Schmitz, 1919)**

**Material examined.** DR 17.07. ♂, 18.08. 2 ♂♂, 04.09. 2 ♂♂, 07.09. ♂, 11.09. ♂, 18.09. 3 ♂♂, 26.09. ♂, 09.10. 3 ♂♂, 10.10. ♂, 13.10. 2 ♂♂, 23.10. 3 ♂♂; HR 22.08. ♂, 11.09. ♂, 14.09. ♂, 22.09. ♂, 03.10. 10 ♂♂, 13.10. 3 ♂♂, 28.10. 4 ♂♂.

**GBIF records.** In Europe this species is primarily recorded in the northern regions, with some records also from Central Europe. https://www.gbif.org/species/1547024.

**Comments.** The first Slovak record of this species was published by Grundmann et al. (2024).

***Megaseliaaequalis* (Wood, 1909)**

**Material examined.** DR 14.09. ♀; HR 11.09. 2 ♂♂.

**GBIF records.** In Europe this species is mainly recorded in the northern regions. https://www.gbif.org/species/1546813.

*Megaseliaalbicans (Wood, 1908)

**Material examined.** HR 28.07. ♂.

**GBIF records.** In Europe this species is primarily recorded in the northern regions, with some records also from Central Europe and the Balkans. https://www.gbif.org/species/1547725.

**Comments.** The larvae of this species are reported to be mycophagous (Durska 2013). It has been reared from false morel (*Gyromitraesculenta* (Pers.) Fr.,) a mushroom of the family Morchellaceae (Disney 1994).

***Megaseliaalbocingulata* (Strobl, 1906)**

**Material examined.** HR 17.07. 2 ♂♂, 18.08. ♂; DR 17.07. ♂, 31.07. ♂, 11.09. ♂.

**GBIF records.** In Europe this species is primarily recorded in the northern regions. https://www.gbif.org/species/1547320.

*Megaseliaanalis (Lundbeck, 1920)

**Material examined.** DR 26.09. ♂.

**GBIF records.** There are only a few records from Finland and Sweden. https://www.gbif.org/species/1548247.

**Comments.** The larval diet is unknown (Durska 2013).

***Megaseliaangularis* (Schmitz, 1924)**

**Material examined.** DR 14.08. ♂, 18.08. 2 ♂♂; HR 17.07. ♂, 18.08. ♂, 22.08. 2 ♂♂.

**GBIF records.** There are only a few records from Finland and Sweden. https://www.gbif.org/species/1546855.

***Megaseliaangusta* (Wood, 1909)**

**Material examined.** DR 28.07. ♂, 09.08. ♂, 28.08. ♂, 04.09. ♀, 11.09. 2 ♂, 14.09. ♂, 18.09. 4 ♂♂, 22.09. 2 ♂♂, 09.10. ♂, 10.10. ♂, 28.10. 2 ♂♂; HR 17.07. ♂, 21.07. 3 ♂♂, 24.07. 12 ♂♂, 28.07. ♂, 07.08. 3 ♂♂, 3 ♀♀, 25.08. ♂, 28.08. 2 ♂♂, 04.09. 3 ♂♂, 07.09. 16 ♂♂, 11.09. ♂, 14.09. ♂, 22.09. ♂, 26.09. ♂, 03.10. 2 ♂♂, 28.10. ♂.

**GBIF records.** In Europe this species is primarily recorded in the northern regions. https://www.gbif.org/species/1547218.

*Megaseliaannulipes (Schmitz, 1921)

Fig. 2

**Figure 2.**
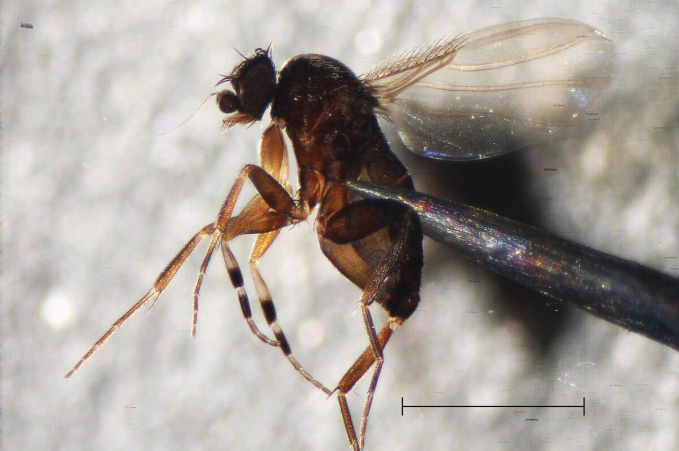
*Megaseliaannulipes* (Schmitz, 1921). This species is easy recognisable by its annulated front tarsi, although little is known about their function. A signalling function in the context of mating behaviour could be conceivable (photograph by www.spessart-fliegen.de). Scale bar 1 mm.

**Material examined.** DR 14.09. ♂.

**GBIF records.** In Europe this species is primarily recorded in the northern regions, with some records also from Central Europe and the Balkans. https://www.gbif.org/species/1546937.

**Comments.** The larva is a parasitoid of spiders. It has been reared from *Moebeliapenicillata* (Westring, 1851), a spider of the family Linyphiidae (Weber et al. 2006).

*Megaseliabasispinata (Lundbeck, 1920)

**Material examined.** DR 09.08. ♂.

**Published records.** This Holarctic-Neotropical species is known from Europe, Macaronesia, and North and South America (Langourov 2004a).

**GBIF records.** In Europe this species is primarily recorded in the northern regions. https://www.gbif.org/species/1548403.

**Comments.** The larval diet is unknown (Durska 2013).

***Megaseliaberndseni* (Schmitz, 1919)**

**Material examined.** HR 26.07. 2 ♂♂.

**GBIF records.** In Europe this species is primarily recorded in the northern regions, with some records also from Central Europe. https://www.gbif.org/species/4516507.

***Megaseliabrevicostalis* (Wood, 1910)**

**Material examined.** DR 18.08. ♂; HR 21.07. ♂, 22.08. ♂, 28.08. ♂.

**GBIF records.** In Europe this species is primarily recorded in the northern regions, with some records also from Central Europe. https://www.gbif.org/species/1547644.

*Megaseliabrevissima Schmitz, 1924

**Material examined.** DR 31.07. ♂.

**GBIF records.** No distribution records are currently shown on the map. https://www.gbif.org/species/1547213.

**Comments.** Identification with Disney (2006a). This species belongs to the *Megaseliabrevior* species complex.

***Megaseliabreviterga* (Lundbeck, 1920)**

**Material examined.** DR 26.07. ♀

**GBIF records.** In Europe this species is primarily recorded in the northern regions. https://www.gbif.org/species/1548369.

**Comments.** The first record was published by Grundmann et al. (2023).

***Megaseliaciliata* (Zetterstedt, 1848)**

**Material examined.** HR 25.08. ♂; 07.09. ♂.

**GBIF records.** In Europe this species is primarily recorded in the northern and Central European regions. https://www.gbif.org/species/1546686.

***Megaseliacinereifrons* (Strobl, 1910)**

**Material examined.** HR 21.07. 2 ♂♂; HR 31.07. ♂.

**GBIF records.** In Europe this species is primarily recorded in the northern regions, with some records also from Central Europe. https://www.gbif.org/species/1548194.

***Megaseliacommuniformis* (Schmitz, 1918)**

**Material examined.** HR 07.08. ♂.

**GBIF records.** There are only few records from Sweden and Germany. https://www.gbif.org/species/1547173.

***Megaseliaconformis* (Wood, 1909)**

**Material examined.** DR 07.09. ♂.

**GBIF records.** In Europe this species is primarily recorded in the northern regions. https://www.gbif.org/species/1548180.

*Megaseliacrassipes (Wood, 1909)

**Material examined.** HR 28.08. ♂.

**GBIF records.** In Europe this species is primarily recorded in the northern regions, with some records also from Central Europe. https://www.gbif.org/species/1546701.

**Comments.** The larval diet is unknown (Durska 2013).

*Megaseliacurvicapilla Schmitz, 1947

**Material examined.** HR 28.08. ♂, HR 04.09. ♂.

**GBIF records.** There are only few records from Finland, Sweden, and Germany. https://www.gbif.org/species/1546685.

**Comments.** The larval diet is unknown (Durska 2013).

*Megaseliadeltomera (Schmitz, 1924)

**Material examined.** HR 21.07. ♂, HR 22.09. ♂.

**GBIF records.** No distribution records are currently shown on the map. https://www.gbif.org/species/1547749.

**Comments.** Identification with Disney (2004).

*Megaseliadensior Schmitz, 1927

**Material examined.** DR 31.07. ♂, 11.09. 2 ♂♂, 22.09. 2 ♂♂, 26.09. ♂, 09.10. ♂; HR 26.07. ♂; 28.07. ♂, 07.08. ♂, 22.08. ♂, 11.09. 2 ♂♂, 14.09. ♂.

**GBIF records.** No distribution records are currently shown on the map. https://www.gbif.org/species/1547247.

**Comments.** This is the eponymous species of the *Megaseliadensior* species complex, which has been revised by Buck and Disney (2001).

***Megaseliaemarginata* (Wood, 1908)**

**Material examined.** DR 21.07. ♂, DR 26.07. ♂; HR 25.08. ♂.

**GBIF records.** In Europe this species is primarily recorded in the northern regions, with some records also from Central Europe. https://www.gbif.org/species/1546757.

***Megaseliaerrata* (Wood, 1912)**

**Material examined.** DR 28.07. ♂, DR 18.08. ♂.

**GBIF records.** In Europe this species is primarily recorded in the northern regions. https://www.gbif.org/species/1548095.

*Megaseliaexcorticata Disney, 2010

**Material examined.** DR 11.09. ♂, 10.10. ♂; HR 26.07. ♂, 07.08. ♂, 18.08. ♂, 13.10. ♂.

**Published records.** In addition to the type locality in Finland, there is only one further record from Germany (Hannig et al. 2023).

**GBIF records.** There are only few records from Finland. https://www.gbif.org/species/11161573.

**Comments.** Identification with Disney (2010a).

*Megaseliafenestralis (Schmitz, 1919)

**Material examined.** DR 21.07. ♂, 14.08. ♂, 25.08. ♂, 28.08. ♂; HR 17.07. ♂.

**GBIF records.** There are only few records from Finland and Sweden. https://www.gbif.org/species/1547966.

**Comments.** The larval diet is unknown (Durska 2013).

***Megaseliaflava* (Fallén, 1823)**

**Material examined.** HR 18.09. ♀.

**GBIF records.** In Europe this species is primarily recorded in the northern regions, with some records also from Central Europe. https://www.gbif.org/species/1547612.

*Megaseliaflavescens (Wood, 1909)

**Material examined.** DR 21.07. ♂.

**GBIF records.** There are only few records from Finland, Sweden, and Britain. https://www.gbif.org/species/1547805.

**Comments.** The larval diet is unknown (Durska 2020).

***Megaseliaflavicans* Schmitz, 1935**

**Material examined.** DR 31.07. ♂.

**GBIF records.** In Europe this species is primarily recorded in the northern regions. https://www.gbif.org/species/1546504.

***Megaseliaframeata* Schmitz, 1927**

**Material examined.** DR 26.09. ♂, 09.10. 2 ♂♂, 23.10. 2 ♂; HR 21.07. ♂, 24.07. 2 ♂, 22.08. ♂, 25.08. 2 ♂, 04.09. ♂, 04.09. ♂, 14.09. ♂, 18.09. 3 ♂♂, 22.09. 3 ♂♂, 26.09. 17 ♂♂, 03.10. 22 ♂♂, 13.10. ♂, 28.10. 3 ♂♂.

**GBIF records.** In Europe this species is primarily recorded in the northern regions, with some records also from Central Europe. https://www.gbif.org/species/1547462.

***Megaseliagiraudii* (Egger, 1862)**

**Material examined.** DR 25.08. ♂, 28.08. ♂, 11.09. ♂, 14.09. ♂, 09.10. 2 ♂♂; HR 17.07. 2 ♂♂, 21.07. ♂, 24.07. ♂, 18.08. 2 ♂♂, 22.08. ♂, 25.08. 2 ♂, 28.08. 2 ♂, 04.09. ♂, 07.09. ♂, 11.09. 5 ♂♂, 26.09. ♂.

**GBIF records.** In Europe this species is primarily recorded in the northern regions. https://www.gbif.org/species/4295479.

***Megaseliagregaria* (Wood, 1910)**

**Material examined.** DR 17.07. ♂.

**GBIF records.** In Europe this species is primarily recorded in the northern regions, with some records also from Central Europe. https://www.gbif.org/species/1546523.

*Megaseliahartfordensis Disney, 1983

**Material examined.** DR 21.07. ♂; HR 14.09. ♂.

**Published records.** In addition to the type locality in England, there is one record from France (Disney and Withers 2011) and one from Germany (Hannig et al. 2023).

**GBIF records.** There is only one record from Britain. https://www.gbif.org/species/1546633.

***Megaseliahilaris* Schmitz, 1927**

**Material examined.** HR 28.08. ♂.

**GBIF records.** In Europe this species is recorded in the northern and central regions. https://www.gbif.org/species/1546935.

*Megaseliaignobilis (Schmitz, 1919)

**Material examined.** DR 17.07. 2 ♂♂, 03.08. ♂, 25.08. ♂, 07.09. ♂, 11.09. ♂, 09.10. ♂; HR 24.07. 2 ♂♂.

**GBIF records.** In Europe this species is primarily recorded in the northern regions, with some records also from Central Europe. https://www.gbif.org/species/1546714.

**Comments.** The larval diet is unknown (Durska 2015, 2020).

*Megaseliaintergeriva Schmitz, 1948

**Material examined.** HR 28.08. ♂.

**Published records.** Identification followed Disney (2010b). This species has been described from Austria. Disney (2010b) mentions finds from Switzerland where flies have been reared from spruce trunks. It is also known from Germany (Hannig et al. 2023).

**GBIF records.** No distribution records are currently shown on the map. https://www.gbif.org/species/1548170.

***Megaseliainvoluta* (Wood, 1910)**

**GBIF records.** In Europe this species is primarily recorded in the northern regions, with some records also from Central Europe and the Balkans. https://www.gbif.org/species/1548490.

**Material examined.** HR 26.07. ♂, 28.07. ♂.

*Megaselialatipalpis (Schmitz, 1921)

**Material examined.** DR 26.07. ♂, 31.07. ♂, 07.09. 2 ♂♂, 11.09. ♂, 14.09. ♂; HR 24.07. ♂, 31.07. ♂, 25.08. ♂, 04.09. ♂, 07.09. 3 ♂, 11.09. ♂.

**Published records.** European species, known from Austria, Balkan Peninsula, Germany, Great Britain, and France (Langourov 2004b).

**GBIF records.** There are only few records from Sweden and Britain. https://www.gbif.org/species/1546846.

***Megaselialedburiensis* Brues, 1915**

*Megaseliasubfuscipes* Schmitz, 1935. Syn.

**Material examined.** HR 03.10. ♂.

**GBIF records.** There are only few records Britain. https://www.gbif.org/species/10139024.

**Comments.** The first Slovak record of this species was published by Grundmann et al. (2024).

***Megaseliamajor* (Wood, 1912)**

**Material examined.** DR 31.07. ♂.

**GBIF records.** There are only a few records from Finland, Sweden, and Britain. https://www.gbif.org/species/1547851.

*Megaseliamalhamensis Disney, 1986

**Material examined.** DR 14.09. ♂; HR 17.07. ♂, 22.08. ♂.

**Published records.** This species has been described from England and is also known from Germany.

**GBIF records.** There are only few records from Finland, Sweden, Germany, and the Balkans. https://www.gbif.org/species/1547856.

**Comments.** Identification with Buck and Disney (2001).

***Megaseliamanicata* (Wood, 1910)**

**Material examined.** DR 11.09. ♂; HR 31.07. ♂, 22.08. ♂, 25.08. ♂, 28.08. 2 ♂♂.

**GBIF records.** In Europe this species is primarily recorded in the northern regions, with some records also from Central Europe. https://www.gbif.org/species/1547792.

**Comments.** The first Slovak record of this species was published by Grundmann et al. (2024).

***Megaseliamaura* (Wood, 1910)**

**Material examined.** DR 13.10. ♂.

**GBIF records.** There are only a few records from Finland, Sweden, and Britain. https://www.gbif.org/species/1546902.

***Megaselianasoni* (Malloch, 1914)**

**Material examined.** HR 22.08. ♂.

**GBIF records.** There are several records from Finland, Sweden, and Britain. https://www.gbif.org/species/4516511.

***Megaselianigra* (Meigen, 1830)**

**Material examined.** HR 26.07. ♂.

**GBIF records.** In Europe this species is primarily recorded in the northern regions. https://www.gbif.org/species/1547368.

***Megaseliapectoralis* (Wood, 1910)**

**Material examined.** DR 13.10. ♂; HR 14.09. ♂, 26.09. ♂.

**GBIF records.** There are several records from Finland, Sweden, and Britain. https://www.gbif.org/species/1546740.

***Megaseliapleuralis* (Wood, 1909)**

**Material examined.** DR 04.09. ♂, 18.09. 2 ♂, 09.10. ♂, 10.10. ♂; HR 17.07. ♂, 31.07. ♂, 18.09. ♂, HR 03.10. ♂.

**GBIF records.** In Europe this species is primarily recorded in the northern regions, with some records also from Central Europe. https://www.gbif.org/species/1548494.

***Megaseliapropinqua* (Wood, 1909)**

**Material examined.** HR 21.07. ♂.

**GBIF records.** There are several records from Finland, Sweden, and Britain. https://www.gbif.org/species/1546995.

***Megaseliapusilla* (Meigen, 1830)**

**Material examined**. DR 21.07. 2 ♂♂, 25.08. ♂, 07.09. 2 ♂♂, 14.09. ♂, 09.10. ♂, 10.10. ♂, 23.10. ♂; HR 22.08. ♂, 28.08. 2 ♂, 04.09. ♂, 09.10. ♂.

**GBIF records.** In Europe this species is primarily recorded in the northern regions, with some records also from Central Europe. https://www.gbif.org/species/1548452.

***Megaseliapygmaea* (Zetterstedt, 1848)**

**Material examined.** DR 03.08. ♂, 09.08. ♂, 11.09. ♂.

**GBIF records.** In Europe, this species is primarily recorded in the northern regions. https://www.gbif.org/species/1547970.

***Megaseliaquadriseta* (Schmitz, 1918)**

**Material examined.** DR 28.07. ♂, 03.08. 2 ♂, 25.08. ♂, 07.09. ♂, 11.09. 3 ♂, 10.10. ♂; HR 21.07. ♂, 14.09. 3 ♂♂, 03.10. ♂, 28.10. 2 ♂♂.

**GBIF records.** In Europe this species is primarily recorded in the northern regions, with two records also from Central Europe. https://www.gbif.org/species/1547785.

***Megaseliarubescens* (Wood, 1912)**

**Material examined.** DR 09.10. ♂; HR 18.08. ♂, 11.09. ♂.

**GBIF records.** There are several records from Sweden and Britain. https://www.gbif.org/species/1547312.

**Comments.** The first Slovak record of this species was published by Grundmann et al. (2024).

***Megaseliarufipes* (Meigen, 1804)**

**Material examined.** DR 11.09. 2 ♂♂, 14.09. ♂.

**GBIF records.** In Europe this species is primarily recorded in the northern regions, with some records also from Central Europe and the Balkans. https://www.gbif.org/species/1547156.

*Megaseliashawi Disney, 2006

**Material examined.** DR 09.10. ♂; HR 28.07. ♂, 04.09. ♂.

**Published records.** Identification with Disney (2006b). This species is a sibling species of the common *Megaseliaframeata*. It is known only from the type locality in Scotland. Meanwhile, *M.shawi* proved to be an overlooked species, which is widely distributed in Europe (BG unpublished). The type specimens have been reared from a dead *Salix* trunk.

**GBIF records.** No distribution records are currently shown on the map. https://www.gbif.org/species/1547691.

*Megaseliaspeiseri Schmitz, 1929

**Material examined.** DR 26.09. ♂, 10.10. ♂.

**Published records.** This species has been recorded as new for the German fauna by Prescher and Bellstedt (1994).

**GBIF records.** There are several records from Sweden, Finland, Denmark, and Germany. https://www.gbif.org/species/1548455.

*Megaseliasubcarpalis (Lundbeck, 1820)

**Material examined.** DR 14.08. ♂, 11.09. 2 ♂, 26.09. ♂; HR 21.07. 2 ♂, 11.09. ♂, 14.09. 2 ♂♂, 26.09. ♂.

**GBIF records.** There are several records from Sweden and Finland. https://www.gbif.org/species/1546628.

**Comments.** The larval diet is unknown (Durska 2013).

*Megaseliasubconvexa (Lundbeck, 1920)

**Material examined.** DR 28.08. ♂, 07.09. ♂; HR 22.08. ♂, 25.08. ♂, 07.09. 2 ♂♂, 11.09. 2 ♂♂.

**Published records.** This species is only known from Denmark and the Netherlands (Schmitz 1941), from Sweden (Bonet et al. 2011), and from Germany (Hannig et al. 2023).

**GBIF records.** There are several records from Sweden, Britain, and Germany. https://www.gbif.org/species/1547641.

***Megaseliasubtumida* (Wood, 1909)**

**Material examined.** HR 24.07. ♀, 28.07. ♀, 31.07. ♀, 07.08. ♀, 07.09. ♀.

**GBIF records.** In Europe this species is primarily recorded in the northern regions, with some records also from Central Europe. https://www.gbif.org/species/1547985.

*Megaseliasurdifrons (Wood, 1909)

**Material examined.** DR 22.09. ♂.

**Published records**. Historical records exist from Denmark, the Netherlands, and Switzerland (Schmitz 1941), while more recent findings have been reported from Norway (Disney 2015) and Germany (Hannig et al. 2023).

**GBIF records.** There are several records from Sweden and Denmark. https://www.gbif.org/species/1547995.

*Megaseliasylvatica (Wood, 1910)

**Material examined.** DR 11.09. ♂, 13.10. ♂.

**GBIF records.** There are several records from Finland, Sweden, and Britain. https://www.gbif.org/species/1547326.

**Comments.** The larvae of this species have been reported to be mycophagous (Durska 2013, 2015, 2020) and have been reared from mushrooms belonging to the families Pleurotaceae and Plutaceae (Disney 1991).

***Megaseliavernalis* (Wood, 1909)**

**Material examined.** HR 03.10. ♂.

**GBIF records.** There are several records from Finland, Sweden, Germany, and Britain. https://www.gbif.org/species/1547053.

**Comments.** The first Slovak record of this species was published by Grundmann et al. (2023).

## ﻿Discussion

This study does not aim to provide a statistically verifiable comparison between the method employed here and commonly used techniques for sampling flying insects. Such a comparison would have required the simultaneous deployment of Malaise traps or yellow pan traps at multiple comparable locations within the zoo. Instead, the primary objective is to demonstrate that the sustainable use of non-target organisms can yield valuable faunistic data. This is particularly relevant for taxa that remain relatively understudied, such as the Phoridae. Nor do we attempt to speculate whether the same species would have been detected using alternative trapping methods. Environmental factors such as vegetation, soil type, microclimate, and the surrounding landscape context exert at least as much influence on species detection as the choice of sampling method itself (Matthews and Matthews 1971). For a comprehensive and detailed analysis of various arthropod sampling techniques, see Schuch et al. (2020).

**Figure 3.**
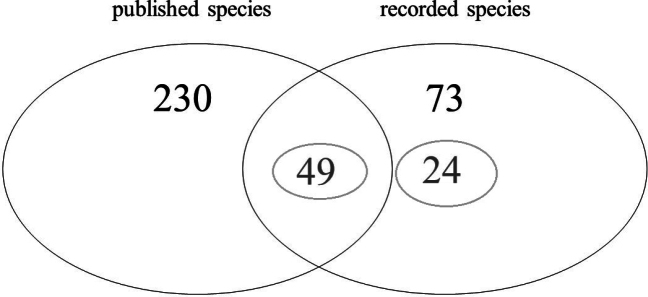
Comparison of the known Slovak phorid fauna (230 species published up to 2024) with the species recorded in the present study (73 species in total, of which 24 are new for Slovakia). The overlapping area represents 49 species, accounting for approximately 21% of the previously documented Slovak phorid fauna.

Depending on the research objective and the methodology applied, investigations involving automated sampling systems typically generate varying amounts of bycatch (non-target organisms) which in most cases remain unanalysed. Only a few projects adopt a holistic approach, aiming to identify and assess as many taxa as possible (Hannig et al. 2023). This study instead seeks to demonstrate that the targeted evaluation of individual, selectively chosen taxa can yield valuable faunistic insights, even in cases where statistical analysis is not feasible. In this study, we present the results obtained using two BG-Sentinel 2 traps combined with CO_2_ as an attractant. A total of 73 species of the family Phoridae – represented by nearly 460 individuals – were recorded from these traps between July and October 2023 at Košice Zoo, located in eastern Slovakia. Of these, 24 species are reported for the first time in the Slovak fauna (Fig. 3), increasing the total number of known phorid species in the country from 230 (Mocek 1997, 2009; Jančík and Disney 2020; Grundmann et al. 2023, 2024) to 254.

For comparison, several other studies on Phoridae have reported markedly different results depending on sample size, trapping effort, and methodology. Grundmann and Kappert (2023) identified 71 species (68 from Malaise traps and three from other collection methods) based on a total of nearly 24,000 individuals. Brown and Hartop (2017) recorded 99 species from approximately 42,000 individuals, while Durska (2009) reported 52 species from a sample of 6,000 individuals. In contrast, the present study recorded 73 species from fewer than 500 individuals, highlighting the potential efficiency of the method used. To the best of the authors’ knowledge, this is the first application of BG-Sentinel 2 traps with CO_2_ for phorid sampling. While this approach may not yield large specimen numbers, it appears to be effective in detecting a high diversity of species, including a significant proportion that are new to the national fauna. Notably, Beuk (2023), using Malaise traps in the Jean Massart Botanic Garden (Auderghem), collected only 126 specimens representing 24 species in 11 genera – further suggesting that even traditional, widely used methods may yield limited results under certain environmental conditions or in short sampling periods. These results support the idea that greater attention should be paid to non-target organisms collected during entomological surveys, especially when using automated or passive trapping systems. While such material is often regarded as waste, it can represent a significant and largely untapped resource for biodiversity studies, particularly in underexplored or hyperdiverse insect groups such as the Phoridae. We therefore encourage researchers to consider incorporating the analysis of bycatch into their study designs whenever feasible. Even if comprehensive identification of all non-target taxa is not possible, targeted examination of selected groups (such as phorid flies) can yield valuable faunistic, ecological, and biogeographical insights. This approach aligns with the broader goals of sustainability and resource efficiency in biodiversity research, maximising the scientific return from existing sampling efforts. Furthermore, the reuse and re-evaluation of previously overlooked material may facilitate cross-disciplinary collaborations between experts focused on different insect taxa. As demonstrated in this study, what was initially collected for mosquito surveillance turned out to be a rich source of novel data on scuttle flies – revealing both new country records and significant species diversity.

## ﻿Conclusions

This study demonstrates that even non-traditional sampling methods, such as BG-Sentinel traps primarily designed for mosquito monitoring, can yield valuable insights into the diversity of non-target insect groups. The discovery of 24 previously unrecorded phorid species for Slovakia emphasises the untapped potential of bycatch material in biodiversity research. This approach not only improves the efficiency of sampling efforts but also opens new avenues for discovering and documenting understudied taxa.

We recommend that future entomological surveys incorporate the analysis of bycatch to enhance the breadth of faunistic data collected. By doing so, researchers can maximise the scientific value of existing collection methods, uncover hidden biodiversity, and foster cross-disciplinary collaboration. Ultimately, the re-evaluation of “unwanted” samples can contribute significantly to our understanding of insect diversity and ecology.

## ﻿Additional information

### Author ORCIDs

Bernd Grundmann https://orcid.org/0009-0007-8478-0188

Katarína Loziaková Peňazziová https://orcid.org/0000-0002-3434-5977

Tomáš Csank https://orcid.org/0000-0003-2055-9293

Laura Mlynárová https://orcid.org/0009-0004-1817-7792

Jozef Oboňa https://orcid.org/0000-0002-1185-658X

### Data availability

All of the data that support the findings of this study are available in the main text.
